# Immunoregulatory effects of AFP domains on monocyte-derived dendritic cell function

**DOI:** 10.1186/1471-2172-12-4

**Published:** 2011-01-17

**Authors:** Agus Setiyono, Akterono D Budiyati, Sigit Purwantomo, Madonna R Anggelia, Ismail Fanany, Gunawan A Wibowo, Indra Bachtiar, Andi Utama, Susan Tai

**Affiliations:** 1 Mochtar Riady Institute for Nanotechnology, Tangerang 15810, Indonesia

## Abstract

**Background:**

Alpha-fetoprotein (AFP) is a tumor-associated glycoprotein that functions in regulation of both ontogenic and oncogenic growth. Recent study showed that AFP can induce apoptosis or impair monocyte-derived dendritic cell (MDDC) function. However, it is still unclear which AFP domain (D-AFP) plays major role in this function.

**Results:**

As expected monocytes cultured in the presence of Granulocyte Macrophage-Colony Stimulating Factor (GM-CSF) and Interleukin-4 (IL-4) developed into MDDC. Up-regulation of HLA-DR and CD11c as well as loss of CD14 molecules could be observed. Full length AFP (FL-AFP), domain 2 AFP (D2-AFP) and D3-AFP, but not D1-AFP, significantly inhibited the expression of HLA-DR^high^/CD11c^high ^and CD80^+^/CD86^high ^molecules. In contrast, CD83 expression was substantially down-regulated in all samples. Expression of CD40 was significantly suppressed by FL-AFP but not by any D-AFPs. Finally, both FL-AFP and D-AFP impaired the MDDC ability to secrete IL-12 (p70).

**Conclusions:**

D2- and D3- but not D1-AFP extensively suppresses the MDDC function. All the recombinant AFP proteins impaired the ability of MDDC to secrete IL-12.

## Background

Human alpha-fetoprotein (AFP) is a tumor-associated fetal glycoprotein that functions in regulation of both ontogenic and oncogenic growth [1]. It consists of 15 disulfide bridges located at positions equivalent to human albumin, leading to a three dimensional structure that is similar to human albumin [2]. One of the biological properties of AFP is its regulatory effects on immune responses. In hepatocellular carcinoma (HCC) patients with high levels of AFP, antigen presenting cells (APCs) are dysfunctional. This leads to the suppression of T- and B-cells response [3]. Dendritic cells (DCs) are the most potent APCs and are important for the initiation of the immune response against pathogens and tumors. In an *in vitro *experiment, Um *et al*. (2004) reported that AFP treatment of DCs reduced the ability of monocyte-derived DC (MDDC) to produce IL-12 and induces apoptosis of MDDC [4]. However, it is still unclear which domain of the AFP plays the important role in apoptosis or impairment of the DC functions.

The aims of this study were to produce recombinant AFP domain (D-AFP) proteins in an *Escherichia coli *expression system and to investigate the immunoregulatory properties of each D-AFP and to compare them to full length (FL)-AFP. Secretion of IL-12 by MDDC after treatment with D-AFP and FL-AFP was also analyzed.

## Results

### Cloning, expression, and purification of alpha-fetoprotein domains

**Figure 1:**
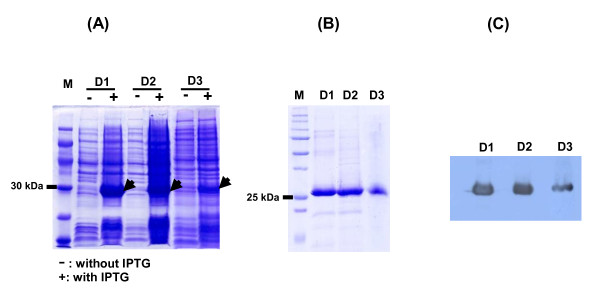
**Purification of the AFP domains**. (A) SDS-PAGE of total lysates without or with beta-gal induction (control and induction were denoted as - or + respectively). Gels were stained with Coomassie blue. M denotes protein marker. (B) Coomassie stained gels of purified proteins from each domain with molecular weight of 24.8 kDa (D1) and 24.4 kDa for D2 and D3. (C) Gel from B) was transferred to the nitrocellulose membrane and blotted with an anti-His antibody.

Several *E. coli *expressing plasmid constructs were made to generate the recombinant AFPs. Recombinant AFP plasmids containing domain 1 and domain 2 were made in accordance with Morinaga et al (1983) [domain 1: amino acids position 1-197, domain 2: amino acids position 198-389], and domain 3 was produced with amino acid positions 357-590 [5]. The N-terminal of recombinant D1-AFP and D2-AFP contain an extra 14 amino acid peptide (MASMTGGQQMGRDP) while D3-AFP contains additional 2 amino acids (MA). To aid the purification process, all recombinant proteins also have 6xHis tag in their C-terminal ends (LEHHHHHH). D-AFPs were successfully expressed by Isopropyl β-D-1-thiogalactopyranoside induction as confirmed by SDS-PAGE and Western blot using anti-His antibody (Figures 1A &1C). They were successfully purified by Ni-NTA column (Figure 1B).

### Impact of the domains on MDDC maturation *in vitro*

**Figure 2:**
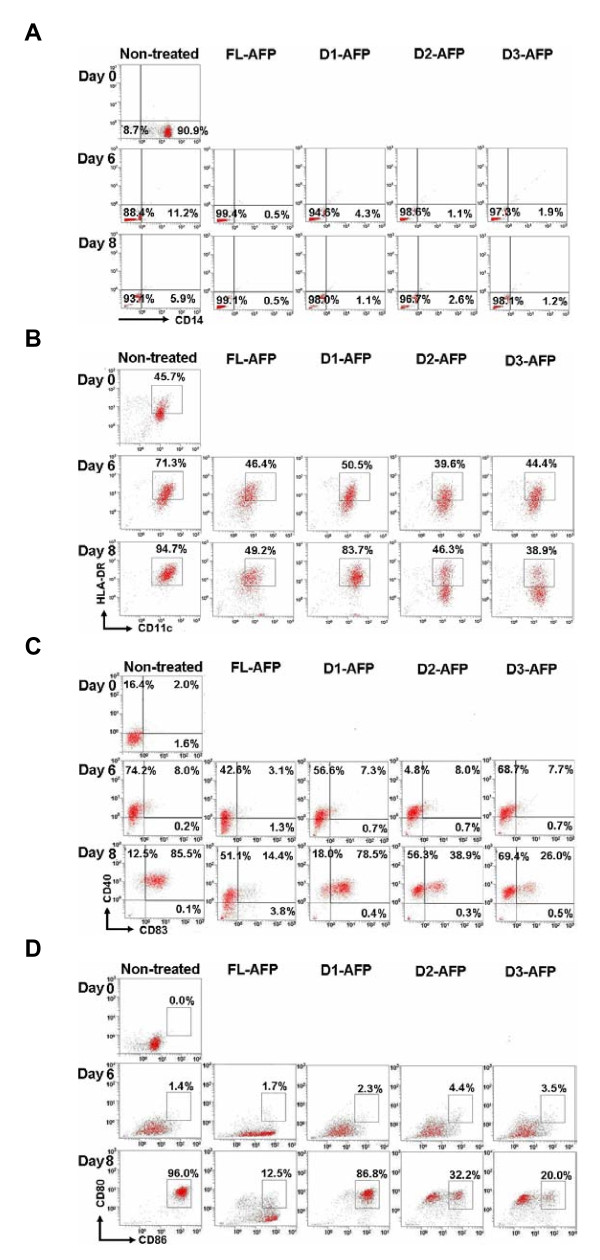
**Effect of AFP domains on MDDC maturation and function *in vitro***. (A) CD14 expression pattern during DC maturation. Examination of surface markers for (B) HLA-DR and CD11c, (C) CD40 and CD83, and (D) CD80 and CD86 to monitor MDDC function.

As shown in Table 1 and Figure 2, freshly isolated monocytes from human PBMC (day 0) were CD14^+^. After stimulation with GM-CSF, IL-4 and LPS, a high expression level of CD83 (day 8) (80.97 ± 2.28) was seen, showing that MDDC was successfully derived from blood monocytes. At the same time, up-regulation of HLA-DR and CD11c as well as loss of CD14 molecules were also observed at day 8 (Figure 2B).

**Table 1:** Comparison of the phenotypic characteristic of MDDC at three stages dependent on treatments

		Surface Marker Expression (%)				
		
		**CD14**^**+**^	**HLA-DR**^**high**^	**CD80**^**+**^	**CD40**^**+**^	**CD83**^**+**^
			**/CD11c**^**high**^	**/CD86**^**high**^		
	Monocytes*	91.03 ± 0.75	42.70 ± 2.04	0.03 ± 0.03	22.83 ± 9.92	2.20 ± 0.70

Non Treated	imMDDC	5.80 ± 2.91	67.47 ± 3.94	1.33 ± 0.10	52.47 ± 14.98	15.83 ± 6.70

	mMDDC	5.17 ± 0.82	91.4 ± 3.12	92.57 ± 1.52	96.30 ± 0.75	80.97 ± 2.28

FL-AFP	imMDDC	1.30 ± 0.60	47.73 ± 4.45	2.47 ± 0.43	38.50 ± 7.30	10.33 ± 2.68

	mMDDC	1.47 ± 0.57	53.70 ± 3.94**	17.67 ± 2.35**	65.57 ± 0.30**	27.17 ± 3.94**

D1-AFP	imMDDC	2.40 ± 1.47	56.27 ± 2.50	3.50 ± 0.79	54.80 ± 11.47	12.30 ± 2.10

	mMDDC	1.87 ± 0.38	73.63 ± 4.76	65.47 ± 7.03	90.07 ± 3.66	59.27 ± 8.90

D2-AFP	imMDDC	1.07 ± 0.17	44.30 ± 2.04	6.37 ± 1.09	63.53 ± 10.02	17.10 ± 3.65

	mMDDC	2.40 ± 0.40	41.57 ± 6.20**	28.20 ± 4.59**	87.63 ± 5.51	34.70 ± 3.43**

D3-AFP	imMDDC	1.90 ± 0.53	48.27 ± 2.28	6.90 ± 1.65	66.30 ± 9.89	21.97 ± 5.88

	mMDDC	2.07 ± 0.09	43.80 ± 6.91**	27.47 ± 4.06**	85.27 ± 4.89	39.27 ± 6.21**

* ImMDDC is immature MDDC. mMDDC is mature MDDC. Generation of each cell is described in the "Methods".

**showed significant differences compare to non-treated (Tukey test, p < 0.05)

**Figure 3:**
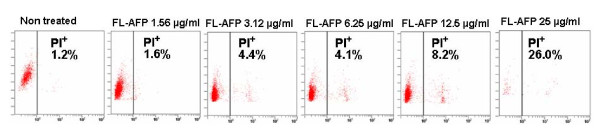
**FL-AFP reduced the viability of MDDC population at high concentrations**. Addition of AFP 12.50 μg/ml or above resulted in a significant cell death of MDDC population based on propidium iodide (PI) assay. PI staining was performed on MDDC culture at day 8, and analyzed with flow cytometry. The viability of MDDC was determined as percentage of negative PI cells. The graphics shown are a representative of three experiments with similar results.

Addition of AFP has previously been shown to induce apoptosis of MMDC [4]. We added a range of AFP concentration between 1.56 μg/ml to 25 μg/ml and observed that MMDC cells start to die at FL-AFP concentration > 12.5 μg/ml (Figure 3). We thus chose the 6.25 μg/ml (0.091 μM) as the working concentration for most of the subsequent experiments. When AFP at concentration 6.25 μg/ml was added to the monocytes culture (day 0), a reduction of the mean expression level of CD83 was observed [from 80.97% to 27.17% in one experiment and 75.6% to 23% in another (Table 1 and 2)]. The same effect was seen for CD80 (Table 2). The AFP immunoregulatory activity is dose-dependent as less suppression was seen at lower AFP concentrations (Table 2). When individual AFP domain proteins were added at 0.091 μM, D1-AFP exhibited little suppressive effect on CD83 (59.27 ± 8.90). In contrast, D2-AFP and D3-AFP showed the same suppressive activities as the full-length AFP protein (Table 1). We concluded that D2-AFP and D3-AFP could significantly block the maturation process of MDDC.

**Table 2:** The viability of MDDC and expression of some surface markers on the mature MDDC treated with FL-AFP at different concentration

	Cell viability (%)		Surface marker expression (%)		
	
	**PI**^**+**^	PI-	**CD80**^**+**^	**CD86**^**+**^	**CD83**^**+**^
Non Treated	1.7 ± 1.0	98.3 ± 1.0	92.3 ± 5.8	99.4 ± 0.6	75.6 ± 24.1

FL-AFP 1.56 μg/ml	2.4 ± 1.7	97.6 ± 1.7	77.1 ± 20.1	98.8 ± 1.1	48.4 ± 21.7

FL-AFP 3.12 μg/ml	3.3 ± 2.2	96.7 ± 2.2	56.3 ± 27.5	99.1 ± 0.4	36.3 ± 21.8

FL-AFP 6.25 μg/ml	3.5 ± 1.1	96.5 ± 1.1	30.9 ± 28.7*	98.7 ± 0.8	23.0 ± 19.8*

FL-AFP 12.5 μg/ml	10.9 ± 5.8	89.1 ± 5.8	31.4 ± 12.6*	92.4 ± 10.2	38.1 ± 14.7

FL-AFP 25 μg/ml	28.2 ± 10.2*	71.8 ± 10.2*	30.0 ± 0.2*	89.7 ± 9.8	2.5 ± 1.6*

* showed significant differences compare to non treated (Tukey test, p < 0.05)

### Impact of the domains on MDDC function *in vitro*

HLA-DR and CD11c were then used for further MDDC characterization. In the presence of 0.091 μM D2-AFP or D3-AFP, MDDC culture failed to up-regulate HLA-DR. In contrast, 0.091 μM D1-AFP treated MDDC culture showed high level of HLA-DR expression. The effect of D2-AFP and D3-AFP on the expression of HLA-DR^high ^was similar to the FL-AFP molecules (Figure 2B). Consistent with the HLA-DR results, expression of CD11c molecule was suppressed at day 8 by full-length AFP (53.70 ± 3.94). The expression of HLA-DR^high^/CD11c^high ^was found to be repressed by D2-AFP (41.57 ± 6.20) and D3-AFP (43.80 ± 6.91) at day 8 (Table 1). This indicated that both D2-AFP and D3-AFP showed a similar suppressive effect to the expression of HLA-DR^high^/CD11c^high ^as the full-length AFP molecules.

In the absence of AFP or domains, CD40 was highly expressed after LPS induction (day 8) as shown in Figure 2. In contrast, addition of 0.091 μM AFP repressed the ability of MDDC to express CD40 as shown in Table 1 (65.57 ± 0.30) at day 8 (bottom panel). In the presence of 0.091 μM D2-AFP or D3-AFP, CD40 expression was altered when compared to non-treated group. However, it was shown that number of CD40^+^/CD83^+ ^cells was reduced by D2-AFP and D3-AFP but not D1-AFP (Figure 2C). We concluded that the expression of CD40^+ ^and CD83^+ ^on MDDC was suppressed when the D2-AFP or D3-AFP was present.

As shown in Figure 2D, after LPS induction, expression of CD80^+^/CD86^high ^was significantly inhibited by 0.091 μM D2-AFP (28.20 ± 4.59) or D3-AFP (27.47 ± 4.06) in a similar effect to the full-length AFP protein (17.67 ± 2.35). In combination with HLA-DR^high ^expression, we concluded that the presence of D2-AFP or D3-AFP is likely to block the ability of MDDC to present antigens. We found that there was no significant difference in the expression of CD80^+^/CD86^high ^when D1-AFP is present during MDDC maturation.

### Effect of FL-AFP or D-AFP on IL-12 secretion

Level of cytokine IL-12 (p70) in supernatant of each MDDC culture was measured using ELISA at day 6 (immature MDDC) and after 48 hrs stimulation with LPS (mature MDDC). Before LPS stimulation, there was very low IL-12 produced by both non-treated and treated group (Table 3). After 48 hrs of LPS stimulation, a significant increase of IL-12 production was detected in the non-treated group but not in the groups treated by FL-AFP or any of the D-AFP samples. These results suggested that both FL-AFP and D-AFP can impair the ability of MDDC to produce IL-12 (p70).

**Table 3:** Production of IL 12 during MDDC maturation process

Treatment	IL12 (pg/ml)	
	
	imMDDC	mMDDC
Non Treated	4.63 ± 2.68	148.13 ± 85.52
FL-AFP	1.60 ± 0.92	0.67 ± 0.38
D1-AFP	1.00 ± 0.58	3.63 ± 2.10
D2-AFP	1.80 ± 1.04	3.67 ± 2.12
D3-AFP	5.03 ± 2.91	1.37 ± 0.79

## Discussion

We investigated the effects of AFP domains on MDDC biology *in vitro*. The study was started by evaluating the effects of AFP full length (FL-AFP) on the viability of MDDC generated from healthy individuals. Using the *E. coli *system and purification procedure as previously reported [5], we could produce the domains of AFP in *E. coli*. In this study, we have delineated the AFP domains that execute the suppressive effect to MDDC maturation. Furthermore, we were interested to see whether after maturation, the MDDC can still function in the presence of the AFP domains. For antigen presentation, the engagement of the T-cell receptor by peptide/MHC complexes (signal 1) is not sufficient to trigger a T cell response, and that ligation of a co-stimulatory receptor (signal 2) is required for T cell activation [6]. In addition, CD40 is also necessary for DC activation [7-10]. Binding CD40 with CD40 ligand will trigger downstream functions of DC such as cytokine secretion. We divided the MDDC functions into the ability to present the antigen and to secrete the cytokine by monitoring the expression of CD14^+^, CD83^+^, HLA-DR^high^/CD11c^high^, CD40^+^/CD83^+^, CD80^+^/CD86^high ^and IL-12 as shown in Table 1 and 2.

Differentiation of monocytes into MDDC was monitored by the expression of CD14 and CD83 molecule. During MDDC maturation, CD14 decreases and conversely, CD83 rapidly increases after addition of LPS. The presence of AFP molecule at the concentration 6.25 μg/ml (0.091 μM) resulted in reduction of CD83 expression. Our results showed that the D2-AFP and D3-AFP retained the suppression effect on CD83 appearance. The suppression effect of D2-AFP and D3-AFP was found to be similar to FL-AFP during the maturation process. As MDDC maturation was repressed, we predict that the function of MDDC would also be suppressed if we added the D2-AFP and D3-AFP.

As shown in Table 1, cells that expressed HLA-DR^high^/CD11c^high^, CD40^+^/CD83^+^, and CD80^+^/CD86^high ^were significantly lower in the presence of D2-AFP and D3-AFP but comparable to the AFP molecules. This finding suggests that these two domains contain the suppression function to MDDC maturation and that D2-AFP or D3-AFP potentially could be used for further study to unravel the role of AFP in the immune system.

Secretion IL-12 in the form of the biologically active p70 molecule could be considered to be one of the most important functions of DCs. IL-12p70 produced by DCs can polarize T cell responses towards CD4+ T helper 1 cells (Th1). Th1 cells can support development of CD8+ cytotoxic T lymphocytes and in turn, can foster an appropriate adaptive immune response to eliminate malignant cells [11]. It has been reported that LPS from *E. coli *could induce MDDC to produce IL-12p70 [12]. However in the presence of the full-length AFP or AFP domains, the production was blocked. These data suggest a differential function of AFP domains in MDDC maturation and cytokine secretion.

AFP is a glycoprotein protein. The recombinant AFP fragments used in our study were expressed in *E. coli*, which does not have a glycosylation system. It is interesting to note that they were still functional in regulating MDDC function similarly to the full-length AFP derived from purified human cord blood serum, which presumably are glycosylated. These results indicated that glycosylation of AFP seems to be unnecessary in their immunoregulatory function. Our result was also supported by a study on the effect of glycosylation on AFP foldability and conformational structure performed by others [13]. Using reversed-phase column HPLC to analyze two AFP variants: glycosylated cord blood-derived AFP and non-glycosylated recombinant AFP purified from transgenic goat milk, they showed that glycosylation is not required for proper protein folding of the human AFP recombinant proteins. They presumed that this is due to the presence of only one single glycosylation site in human AFP [2] and glycosylation does not have a significant impact on the hydrophobicity or disulfide conformation of the molecule.

It has been reported that DC can be transfected or pulsed with antigens to enhance cytotoxic lymphocyte (CTL) responses [14]. In the context of DC-based immunotherapy, AFP has been considered as a candidate antigen to elicit effective tumor rejection [15,16] but its suppressive activities on DC function have precluded its implementation. Our results indicated that individual AFP domains have differential effects on MDDC maturation and function with D1-AFP having the least suppressive activities. D1-AFP can thus potentially be used as an antigen to pulse MDDC to enhance CTL response against AFP. However, further investigations *in vitro *and *in vivo *are still needed to test this idea.

## Conclusions

In conclusion, the AFP domains indeed have differential immunoregulatory function. In the context of MDDC maturation process, D2-AFP and D3-AFP have similar suppressive function as the full-length AFP molecule. As D1-AFP has no effect on DC function, it is a prime candidate for use in a DC-based immunotherapy.

## Methods

### Cloning and expression of the AFP domains

A complete AFP cDNA including its 5'-untranslated region (5'-UTR) was synthesized by reverse-transcription polymerase chain reaction (RT-PCR) from HepG2 total RNA. RT-PCR was performed using Access Quick RT-PCR System (Promega, Wisconsin, USA) with specific AFP primers (forward primer F1: 5'-CTAAGGATCCATGAAGTGGGTGGAATC-3' and reverse primer R3: 5'- GAGAATTCTTAAACTCCCAAAGCAGCACGAG-3'). The resulting fragment was directly cloned into the TOPO 2.1 vector (Invitrogen, CA, USA). The pET21b clones containing D1 and D2 were verified by sequencing. All the fusion proteins contain 14 amino acid (aa) at their N-terminal ends (MASMTGGQQMGRDP) and 8 aa at their C-terminal ends (LEHHHHHH) derived from pET21b vector. For D3 construct, pET21d was used as a backbone (*Nco*I/*Xho*I). *E. coli *DH5α and BL21 (DE3) were utilized for maintenance and expression, respectively. Expression of proteins was induced by Isopropyl β-D-1-thiogalactopyranoside (IPTG) and confirmed by ECL™ Western Blotting Detection Systems (GE Healthcare, Uppsala, Sweden) using anti-His antibody according to manufacturer's protocol.

### Isolation, refolding, and purification

Recombinant D-AFPs were purified based on previous report [5] with some modifications. Frozen cell pellet from 200 ml culture was resuspended in 1 ml buffer B (0.02 M Tris-Cl, 0.3 M NaCl, pH 8.0). The cell suspension was sonicated on an ultrasonic disintegrator high intensity ultrasonic processor 750 W series (Sonics and Materials, Danbury, USA) (12 × 5s, 4°C) and centrifuged (12,000 *g*, 10 min, 4°C). The pellet was resuspended in 1.6 ml buffer B and centrifuged (12,000 *g*, 10 min, 4°C). This procedure was repeated five times. 1.3 ml buffer A (0.1 M Tris-Cl buffer, 6 M guanidine chlorides, pH 8.0) was used to wash bacterial inclusion bodies continued with sonication (12 × 5s, 4°C) and centrifuged (12,000 *g*, 30 min, 4°C). The supernatant was collected and filtered with a Millex-HV PVDF 0.45 μm (Millipore, Massachusetts, USA) and applied onto 2 ml Ni-NTA superflow column (Invitrogen, Carlsbad, USA). The sorbent was washed consecutively with 3.3 ml buffer A and 3.3 ml buffer C (0.05 M Tris-Cl, 6 M Urea, 0.4 M NaCl, and 0.02 M Imidazole, pH 8.0). The protein was eluted in 2 ml buffer D (0.05 M Tris-HCl, 6 M Urea, 0.4 M NaCl, and 0.3 M Imidazole, pH 8.0). Each fraction from column was collected and analyzed by SDS-PAGE. The D fraction was further concentrated on Amicon stirred cell supplied with a PM-10 membrane (MW cutoff 10 kDa) (Millipore, Massachusetts, USA), and clarified through a Millex-HV PVDF 0.45 μm membrane filter.

Beta-mercaptoethanol was added at the final concentration of 0.1 M and incubated for 1 h at room temperature with continuously stirring. The reduced protein was mixed with 60 ml cold buffer E (0.1 M Tris-HCl, 0.5 M NaCl, 2 mM EDTA, pH 8.0) for 48 h and dialyzed consecutively against buffer E (3L, 48h, 4°C) and buffer F (0.01 M Tris-HCl, 0.15 M NaCl, pH 8.0; 3L, 24h, 4°C). The dialyzed protein was concentrated in an Amicon stirred cell supplied with a PM-10 membrane (MW cutoff 10 kDa) and passed through a Superdex™ 200 10/30 column (Amersham Pharmacia Biotech) with buffer F. The purified protein was analyzed by SDS PAGE.

### Isolation of peripheral blood mononuclear cell (PBMC) from blood

The total of 198 ml blood used in this study was obtained from 8 healthy volunteers with the age of 25 to 35 years old. Informed consent was obtained and the ethical approval was granted from Committee on Health Research Ethics of the Mochtar Riady Institute for Nanotechnology. To obtain monocytes as progenitor cell for generating MDDC, we used vacutainer^® ^with heparin (BD vacutainer) during the phlebotomy procedure. PBMC were then prepared using Ficoll-hypaque (GE Health Care, UK) density gradient separation of phlebotomy products.

### Generation of monocyte-derived dendritic cell (MDDC)

MDDC were generated as described previously [17] with some modifications. In brief, PBMC were adhered to 12 well culture plates with density 1 × 10^6 ^ml^-1 ^for 30 min at 37°C with 5% CO_2_. The non adherent cells were removed by gentle wash and the adherent cells were then cultured in complete medium [RPMI 1640 (Sigma) supplemented with 10% heat-inactivated fetal bovine serum (Gibco), and contained rh-GM-CSF (800 U/ml, BD Bioscience Pharmingen) and rh-IL-4 (1000 U/ml, BD Bioscience Pharmingen]. Where indicated, purified human cord blood AFP (FL-AFP, purity > 95%; Monobind method; Lee Biosolutions, Inc) or each D-AFP was added at day 0 for both immature and mature MDDC stage. Culture was fed with complete medium on day 3 and day 5. The immature MDDC analysis was performed at day 6 by collecting the non-adherent cell from each group. Mature MDDC were obtained by transferring the non-adherent cell to another fresh 12 well plates and LPS 500ng/ml (Sigma Aldrich) was then added to induce MDDC maturation. Forty-eight hours later, the non-adherent cell from each group were collected and used for analysis. Each culture supernatant was stored at -20°C for assessment of secreted cytokine within 1 month later.

### Analysis of MDDC surface markers and viability

Monocytes, immature and mature MDDC were stained with CD14 PE (M5E2), HLA-DR FITC (G46-6), CD11c-PE (B-ly6), CD86-PE (2331 FUN-1), CD80-FITC (L307.4), CD40 FITC (5C3), CD83-PE (Hb15e) and relevant isotype control (BD Bioscience Pharmingen), for 30 minutes at 4°C. The cells were then washed with PBS containing 1% FBS. For cell viability determination, cells were stained with Propidium Iodide (PI) at final concentration 1 μg/ml. The cells were then analyzed within 24 hrs on FACS Epic Altra (Beckman Coulter) and gated according to their size (forward light scatter) and granularity (side light scatter).

### Quantitative measurement of secreted IL-12 by enzyme-linked immunosorbent assay (ELISA)

To observe the ability of mature MDDC to produce IL-12, stored supernatants from whether immature or mature MDDC cultures were thawed and analyzed with the Human IL-12(p70) ELISA Set (BD Bioscience) according to the manufacturer's instruction with detection range between 7.8-500 pg/ml.

### Statistical analysis

The DC surface marker expression was analyzed using EXPO32 ADC Analysis Software (Beckman Coulter). One way ANOVA (Tukey test) was used to compare more than two independent groups.

## Authors' contributions

AS participated in experimental design and implementation. ADB was involved in generation of DC, Flow cytometry analysis, ELISA, and statistical analysis SP was involved in domains-AFP production experimental technique. IB was involved in domains-AFP purification experimental technique. MRA was involved in generation of DC, Flow cytometry analysis, ELISA, and statistical analysis. IF contributed in domains-AFP production. GA carried out domains-AFP purification. AU was involved in experimental technique. ST conceived the study and coordination. All authors read and approved the final manuscript.
